# Correction: Deep learning-based text generation for plant phenotyping and precision agriculture

**DOI:** 10.3389/fpls.2025.1648292

**Published:** 2025-07-10

**Authors:** Li Zhu, Long Tang, Shan Ren

**Affiliations:** ^1^ School of Computer Science, Guangzhou Maritime University, Guangzhou, Guangdong, China; ^2^ Hubei University of Economics, Wuhan, China; ^3^ Hebei Academy of Fine Arts, Shijiazhuang, Hebei, China; ^4^ Hanyang University, Ansan-si, Gyeonggi-do, Republic of Korea

**Keywords:** plant phenotyping, deep learning, generative model, biologically-constrained optimization, precision agriculture

Author “Shan Ren” was assigned as corresponding author. The correct corresponding author is “Long Tang”.

The original version of this article has been updated.

